# Antimicrobial peptides and gut microbiota in homeostasis and pathology

**DOI:** 10.1002/emmm.201201773

**Published:** 2013-08-23

**Authors:** Maureen J Ostaff, Eduard Friedrich Stange, Jan Wehkamp

**Affiliations:** 1 Dr. Margarete Fischer-Bosch-Institute of Clinical Pharmacology, Stuttgart, Germany and University of TuebingenGermany; 2 Department of Gastroenterology, Robert Bosch HospitalStuttgart, Germany

**Keywords:** antimicrobial peptides, defensin, epithelial differentiation, intestinal homeostasis, microbiota

## Abstract

We survive because we adapted to a world of microorganisms. All our epithelial surfaces participate in keeping up an effective barrier against microbes while not initiating ongoing inflammatory processes and risking collateral damage to the host. Major players in this scenario are antimicrobial peptides (AMPs). Such broad-spectrum innate antibiotics are in part produced by specialized cells but also widely sourced from all epithelia as well as circulating inflammatory cells. AMPs belong to an ancient defense system found in all organisms and participated in a preservative co-evolution with a complex microbiome. Particularly interesting interactions between host barrier and microbiota can be found in the gut. The intestinal cell lining not only has to maintain a tightly regulated homeostasis during its high-throughput regeneration, but also a balanced relationship towards an extreme number of mutualistic or commensal inhabitants. Recent research suggests that advancing our understanding of the circumstances of such balanced and sometimes imbalanced interactions between gut microbiota and host AMPs should have therapeutic implications for different intestinal disorders.

## Introduction

We humans, like every other multi-cellular organism, are in a constant race to maintain, renew and replace our epithelial tissues. Mostly, this is not a fair fight as our cells are not only threatened by time itself but often challenged with environmental attacks, such as UV rays, oxidative stress or, of particular note, microbes. The latter not always explicitly classify as a threat since they can deliver beneficial properties to the host or leastwise bring no harm. Specific gut bacteria have for instance been associated with improvement of digestion, absorption, vitamin synthesis and the inhibition of pathogen growth (Saulnier et al, 2009), which classifies them as mutualistic inhabitants. But even when considered friendly, or at least commensal, their population needs to be tightly controlled to ensure it stays benevolent rather than taking advantage of a debilitated host (Eberl, 2010). *Albeit* every human epithelial interface has its own battles to fight, the intestinal tract provides an exceptionally striking situation. Its epithelial renewal rate outruns every other tissue in our body (Gregorieff & Clevers, 2005), calling for an especially controlled and efficient regenerative balance. Along the way, it is constantly confronted with an immensely complex ecology of microorganisms, which again, demands a particularly well-balanced homeostasis. All together up to 10^13^ to 10^14^ microorganisms are harboured in the digestive tract of a typical person, corresponding to a mass of about 1–2 kg and by far more cells than the human body. Up to 1000 different species make up the community, which contains mainly bacteria but can also include some eucaryotes, viruses and archaea. The intestinal colonization starts immediately during birth and soon after, the intestinal lumen is hosting a diverse ecosystem. The microbial composition and distribution of the menagerie varies with age, state of health, residence and potentially diet (Lozupone et al, 2012). Present numbers and types also dramatically vary by intestinal region. Due to the bactericidal properties of the gastric and also bile acids, the stomach and proximal small intestine contain only few microorganisms. Patients with reduced gastric acid levels (achlorhydria or hypochlorhydria for example) may suffer from bacterial overgrowth and subsequent inflammatory or malignant complications (Friis-Hansen, 2006; Husebye, 2005; Naylor & Axon, 2003). With increasing distance from the stomach and less acidic pHs however, bacterial numbers are rising. The ileum for example contains a fairly large amount of aerobic and anaerobic bacteria such as *Enterobacter* and *Lactobacilli*. The most densely populated intestinal region is found in the colon with numbers of up to 10^12^ bacteria per gram of intestinal content and predominately anaerobic representatives such as *Bacteroides, Bifidobacteria, Fusobacteria*, *Clostridium* and *Peptostreptococci* (Sears, 2005). Most intestinal bacteria belong to the phyla *Bacteriodetes* or *Firmicutes* but their composition varies locally and is dependent on the present immune defense strategies as determined by the host's genetic make-up and environmental aspects, *e.g*. the individual nutritional life style.

Early on, gut microbes have been implicated in the development of the intestinal morphology and function (Thompson & Trexler, 1971) based on evidence from germ-free or gnotobiotic animal models. An influence of bacteria has also been studied in the development of Peyer's patches and other gut-associated lymphoid tissue as well as in epithelial cell renewal rates (Heitman et al, 1980; Sun et al, 2004; Yamanaka et al, 2003). It is therefore easily imaginable how the intestinal regenerative balance depends on the epithelium's homeostasis towards its microbiota. This relationship though is by far not restricted to a one-sided influence of the former on the latter, but rather dual. Epithelial innate antibiotic effector molecules, termed antimicrobial peptides (AMPs), can fend off ingested pathogens but also shape and control the composition of commensal inhabitants. In humans, AMPs are expressed both broadly and in specialized cells as well as inducible or constitutively. They represent an ancient or primitive defense mechanism found in virtually every multi-cellular organism. In non-vertebrates, where they often act as the major form of protection against microbes, AMPs have been intensively studied even though many questions still remain unanswered. In humans and other mammals, they also have gained acknowledgement as important immune system contributors and potential future drug candidates (Brogden & Brogden, 2011; Wiesner & Vilcinskas, 2010). Our antimicrobial defense system has undergone an extensive co-evolution with a diverse world of microorganisms. Such long-standing interactions established multiple mechanisms, which ensure a commensal or even better a mutualistic co-existence in the gut (Neish, 2009). Different protein families feature AMPs with the most prominent ones in the intestinal tract represented by defensins, cathelicidins (*e.g*. LL-37), C-type lectins (such as the regenerating islet-derived protein (REG) family), ribonucleases (RNases) and S100 proteins (*e.g*. calprotectin). All these families, as well their most important members have been extensively covered in many excellent reviews (Bevins, 2003; Gallo & Hooper, 2012; Harder et al, 2007; Lai & Gallo, 2009) and are therefore not further described in detail. We will nonetheless later on introduce the group of defensins as central AMPs of the gut, since their mechanisms of action, expression and functions are of particular importance for following discussions.

The recent advances in the field of intestinal AMPs continuously highlight their important role in regulating the gut microbial community while ensuring a beneficial homeostasis at the intestinal barrier (Salzman et al, 2010). One well established example in this context are the inflammation promoting consequences of reduced antimicrobial defenses, as they can be seen in chronic inflammatory bowel diseases (IBD) especially Crohn's disease (CD) (Wehkamp et al, 2008). A new ramification is additionally presented by gut bacterial translocation in liver cirrhosis, which could recently additionally be linked to reduced antimicrobial defense in a rodent model (Teltschik et al, 2011). In parallel, scientific advances in current mechanistic studies have revealed novel antimicrobial modes of actions that nicely illustrate their complex role in host protection. The newly identified defense strategies included a formation of bacteria trapping net structures (Chu et al, 2012), but also an environmental dependent activity activation (Schroeder et al, 2011b) as a newly discovered biological principle. Supplementary, major proceedings in our understanding of the checks and balance systems within epithelial proliferative gut networks provide new views on different intestinal disorders. Our group could, for instance, elucidate an involvement of disturbed Wnt signalling, which is crucial for epithelial proliferation but also Paneth cell antimicrobial function, in chronic small intestinal inflammation (Koslowski et al, 2012; Wehkamp et al, 2007). Moreover, we also reported an influence of gut bacteria regarding the expression of transcription factors controlling epithelial secretory lineage decision making as well as Goblet cell differentiation (Becker et al, 2013).

**Glossary**

**Commensalism**

A form of symbiosis when one organism profits from another without bringing any subsequent harm. In the gut, microorganisms, mainly bacteria, are commensal when they benefit from the host, *e.g*. by profiting from the available food sources, without negatively influencing it.

**Dysbiosis**

Defines a perturbation in the community of microbiota. This most often refers to a shift in the phyla composition of microbiota but can also relate to changes on the species level.

**Gnotobiotic animal models**

Model systems which house a defined microbiome. This technically also includes germ-free animals as the status of their microbiota is defined. It mostly refers to different mono-or multi-associated models. Gnotobiotic animals are normally reared sterile and then confronted with specific microbiota, single strands or defined communities, to study the relationship between the host and its potential symbionts or pathogens.

**Gut microbiome**

The gut microbiome represents the millions of microbial genes of an ample and diverse co-evolved ecosystem of gut microbiota in our intestine.

**Gut microbiota**

A community of different microorganisms in the digestive tract. In humans it represents the largest reservoir of microorganisms and is made up by all together 10^13^ to 10^14^ microbial cells of which bacteria make up the largest part.

**Gut pathobionts**

Originally commensal or mutualistic gut inhabitants, which, due to a change in environment or any level within the host–microbiota relationship develop pathogenic potential and become pathobiontic.

**Homeostasis**

A status of relative stability where a system controls its internal properties and environment. In a living organism, regulatory mechanisms aim at maintaining different parameters at a constant level over potentially wide reaching variations, allowing it to function in a broad range of conditions.

**Mutualism**

Existence of a symbiotic relationship between different organisms in which both reciprocally profit from the situation. In the gut, microorganisms classify as mutualistic when they, in addition to being commensal, also have beneficial properties for the host, *e.g*. the ability to break down otherwise unavailable nutrients thereby improving digestion.

In this review, we therefore aim at discussing the importance of a well-balanced host microbe relationship in the context of intestinal epithelial homeostasis. We will feature several mechanisms that insure a beneficial interaction but nonetheless apologize in advance for not possibly being able to cover all the excellent papers in this very vast field. Due to the complex nature of the topic, we will mainly focus on the role of epithelial antimicrobial defenses, in particular defensins. In addition, we will highlight the influence of microbiota on homeostatic signalling and vice versa at the interface of intestinal epithelia.

## Mastering microbiota

**Figure 1:**
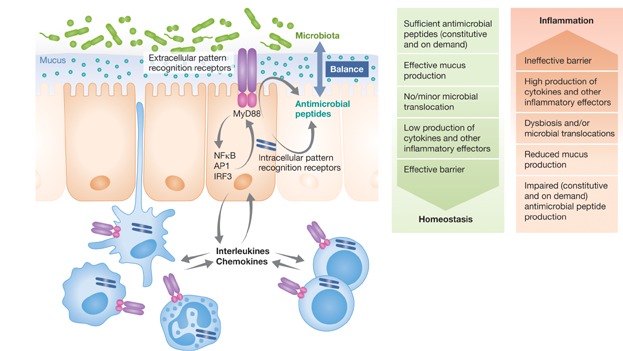
The relationship between resident microbiota and epithelial barrier functions is characterized by a delicate homeostasis Maturated epithelial cells provide not only a physical shield against the luminal content; they also generate potent biological effectors which help to control intestinal microbiota and keep the epithelial adjacent mucus barrier quite sterile. Among these, various AMPs sourced from all epithelia are crucial in maintaining a beneficial homeostasis in the gut. AMPs are in part constitutively expressed but can also be induced by PRR-activated signalling cascades after stimulation with microbial patterns.

Long-standing interactions during our evolution generated a homeostatic and mutualistic relationship between the host and its microbes. While the intestinal symbionts enjoy a more or less ideal habitat with a constant temperature and a continuous supply of nutrients, the human organism also receives considerable benefits, *e.g*. the synthesis of certain vitamins or the breakdown of otherwise indigestible nutritional components. The difference between this fairly peaceful homeostatic host–microbe coexistence and the harmful interactions with pathogens during infections, is however critically dependent on the fact that our intestinal mucosa can hold mutualistic inhabitants at bay. The famous saying ‘good fences make good neighbours’ describes this scenario nicely, as commensal microbiota can also play an important or even central role in the emergence of illnesses which will be discussed in later chapters. The normally well-balanced host–microbe coexistence depends on a complex and multileveled intestinal barrier. The epithelium, as the outermost single cell layer holds an especially critical position. It forms the first line of defense and performs a variety of protective tasks to secure the homeostasis towards symbiotic inhabitants and to avoid invasion with potential pathogens. Intestinal epithelia employ different protective mechanisms that form an intricate innate immune strategy network. Intestinal epithelia probe the resident gut microbiota with pattern recognition receptors (PRR). Those receptors, unlike adaptive immune receptors, require no segmental gene rearrangement and recognize microbes by essential and highly conserved ‘pathogen-associated molecular patterns’ (PAMPs) or ‘microbe-associated molecular patterns’ (MAMPs) (Didierlaurent et al, 2002). The latter term has been proposed since the molecular PAMP motifs, which are recognized by PRRs can also be shared by commensal microorganisms. The M/PAMP-PRR involving way of monitoring gut microbiota has been proven to be crucial for a healthy intestinal barrier (Lee et al, 2006; Rakoff-Nahoum et al, 2004). When stimulated with their respective ligand, PRRs induce a rapid and continuous first line of defense. This includes the production and release of AMPs as well as coordinative signalling molecules and mucins (Cario, 2005; Lievin-Le & Servin, 2006) (Fig 1). Prominent intestinal PRR include the constitutively or inducibly expressed transmembranous surface ‘Toll like receptors’ (TLR's) found on all gut cells, and the intracellular ‘Nucleotide-binding oligomerization domain containing molecules’ (NODs), amongst others (reviewed in Lavelle et al, 2010). The cellular responses following PRR activation are mediated by different signalling molecules and cascades, *e.g*. ‘Myeloid differentiation primary response gene’ (MyD)88, mitogen-activated protein kinases (MAPK) and ‘nuclear factor kappa-light-chain-enhancer of activated B cells’ (NF-κB) signalling. The activated defense program aims at eliminating a potential hazard and can lead to subsequent chemokine-interceded recruitment of acute inflammatory cells and further fostering of NF-κB mediated responses (Santaolalla & Abreu, 2012). In recent years, studies on either the function of a specific PRR or the general intestinal innate recognition machinery have extended our understanding of barrier defense. The major responsibilities have thereby shifted from a short time battle scenario in case of pathogen challenges to a more complex picture with a focus on a sustainable homeostasis towards microbial symbionts. In this framework, it is clear that PRRs must allow a certain amount of tolerance towards the presence of commensal or mutualistic gut microbiota to avoid a constant over- activation. One strategy in promoting a low basal activity is to limit the expression of PRRs. For instance in the human colon, TLR3 (recognizing dsRNA associated with viral infection) and TLR5 (which senses bacterial flagellin) are abundantly expressed, whereas TLR2 (which can be stimulated by different lipopetide agonists (reviewed in Cario, 2008) and TLR4 (which recognises LPS) expression is low in a healthy gut but can be induced (Cario, 2010; Hausmann et al, 2002). An additional example for selective epithelial expression of PRRs is the intracellular, muramyldipeptide sensing ‘Nucleotide-binding oligomerization domain containing molecule’ (NOD)2, which is found in small intestinal epithelia not exclusively but predominantly in AMP producing Paneth cells (Lala et al, 2003). A second crucial factor in dampening PRR mediated inflammatory signalling, is the presence of a sophisticated mucus barrier. Small intestinal epithelia are covered by a single unattached layer of mucus, while the colonic situation exhibits two distinct mucus zones with the inner layer attached and the outer lower density zone remaining unattached (reviewed by Johansson et al, 2011). In the colon, the outer mucus layer constitutes a habitat for commensal bacteria, whereas the inner one functions as a rather sterile seal and is renewed continuously within 1 h (at least in mice) by surface goblet cells (Johansson, 2012). This mucus overlay is not only a physical shield; it retains epithelial produced antimicrobials and thereby provides a competent first line of defense against microbial attachment and invasion (Meyer-Hoffert et al, 2008). To a certain extent, the intestinal lining nonetheless has to come in contact with resident microbiota, as they are essential for different physiological processes. Using germ-free or gnotobiotic animal models as early as 1961, the presence of gut microbiota were already implicated in the development of a proper epithelial architecture (Gordon & Bruckner-Kardoss, 1961a,1961b; Heneghan, 1965). The importance of a somewhat continuous PRR signalling activity, even though it might be low, becomes evident in mice with an epithelial loss of MyD88. The intestinal tissue specific knock out (ko) of this important cytoplasmatic TLR signalling compound leads for example to impaired antimicrobial activity and diminished levels of mucin-2 (the major mucus component), cumulating in differences in gut microbial composition, a greater number of mucus-associated bacteria, translocation events and increased colitis susceptibility (Frantz et al, 2012). A previous study on the influence of MyD88 furthermore focused specifically on small intestinal Paneth cells. Therein, this PRR signalling compound proved to be essential in limiting bacterial penetration by triggering a complex antimicrobial program (Vaishnava et al, 2008). Recently the importance of a well-adjusted interplay between gut bacteria and host innate defenses has also gained awareness in obesity and obesity related metabolic disorders such as diet induced insulin resistance (reviewed in Harris et al, 2012). An especially intriguing example is provided by a study using TLR2 ko mice. Under germfree condition, the absence of this PRR receptor protects against obesity-induced insulin resistance. However, when housed under normal circumstances, the genetic benefit is lost; moreover it is reversed to an increased susceptibility. Loss of TLR2 in intestinal epithelia and subsequently diminished innate defenses preceded changes in the gut microbial composition and creating a menagerie which promoted diminished insulin sensitivity in the host, while overwriting the initial host genetic advantage in this setting (Caricilli et al, 2011).

Such *in vivo* models on epithelial innate immune signalling support a crucial role of basal PRR activation to maintain a beneficial homeostasis towards gut bacteria. They illustrate how a lack of continuous innate defense might promote a switch from a commensal and/or mutualistic relationship towards a pathobiontic character within the gut microbial community.

## Gut defensins

Intestinal epithelia generate specific and competent defensin weapon arsenals with activity against bacteria, viruses, protozoa and fungi reflecting the different threats and challenges, which are met at the mucosa. Members of both groups of gut defensins, α- and β-defensins, are at least in part dependent on innate immune PRRs either for their transcriptional induction or their secretion. In the small intestine, specialized Paneth cells, located at the bottom of crypts of Lieberkühn, store and secrete various antimicrobial effectors (*e.g*. lysozyme, phospholipase A2 group IIA (sPLA2, also known as PLA2G2A) or REGIIIα) (Clevers & Bevins, 2013; George et al, 2008) but their most abundant products are the α-defensins human defensin (HD)5 (gene name DEFA5) and HD6 (gene name DEFA6). HDs exhibit a 3–4 kDa small conserved amphipathic structure with cationic and hydrophobic residues. While HD6 so far only exhibited little bactericidal potential *in vitro* but employs additional defense strategies, which will be discussed in the next chapter, investigations on HD5 exposed an effective killing capacity against various bacteria (Ericksen et al, 2005). Mechanistic studies could also show that an artificial D-enantiomer version of HD5 shows significantly less activity than the native L-form isomer in killing *Staphylococcus aureus*, but equally bactericidal potential against *Escherichia coli* (Wei et al, 2009), revealing an unexpected functional complexity. Both Paneth cell α-defenins also seem to have antiviral activity (Doss et al, 2009; Klotman & Chang, 2006; Wang et al, 2013) but, depending on the setting, might also increase infectivity of certain viruses (Klotman et al, 2008). Furthermore, for HD5, anti-parasitic activity has been reported (Leitch & Ceballos, 2009). The expression of both Paneth cell α-defensins is controlled by different signalling pathways and may depend on an involvement of intracellular NOD2 (also known as CARD15) and the β-catenin dependent Wnt signalling cascade (Koslowski et al, 2009a). The small intestinal subgroup of CD (an IBD which will be discussed in detail below) is linked to major impairments in Paneth cell function and particularly low levels of both HD5 and HD6 (Wehkamp & Stange, 2010). This clinical relevance already indicates their decisive role in gut homeostasis and labels them as promising putative targets for future therapeutic interventions. A second defensin group found in gut epithelial cells, are β-defensins. Different from the Paneth cell restricted HD5 and HD6, with which they share their small size and cationic character, β-defensins are present in a variety of epithelial cells, including enterocytes. While the human β-defensin (HBD) 1 (also known by its gene name *DEFB101*) is constitutively expressed, others, like HBD2 (gene name *DEFB4*) show pathogen and/or inflammation dependent upregulation (Kubler et al, 2009; Ogushi et al, 2001a; Zilbauer et al, 2005, 2010) while also being inducible by probiotic bacteria (Schlee et al, 2007, 2008). Interestingly, HBDs are genetically clustered in a copy-number (CN) or gene dosage variable region with a diploid number of up to 12. CN divergence but also other sequence variations can be important for their level of gene expression (Groth et al, 2010). In a recent worldwide study, Hardwick et al could show how the respective cluster contains rapidly evolving noncoding regulatory sequences. These sites for example can influence the general transcriptional as well as cytokine responsiveness of *DEFB103* (also known as HBD3), an inducible AMP with important antibacterial and antiviral activity against many pathogens, which threaten epithelia. In East Asian individuals, variations resulting in a high HBD3 level were common and possibly linked to the geographically elevated and likely selection-driving incidence of influenza (Hardwick et al, 2011). Even though the work by Hardwick et al specifically focused on HBD3 in a distinct tissue setting, it is a striking example how the level of β-defensin inducibility can have important health related consequences. Another example is provided by colonic CD, which is associated with diminished on demand upregulation and secretion of HBD2 (Wehkamp et al, 2003a, 2008; Zilbauer et al, 2010). Data on β-defensin CN variations (CNV) are however ambiguous in this setting. Earlier evidence on low *DEFB4* copy numbers potentially underlying the impairment were not confirmed (Aldhous et al, 2009; Bentley et al, 2010; Fellermann et al, 2006). Different from the inducible β-defensins, HBD1 appears to be stable during infection or inflammation, but again shows general low mRNA in colonic CD. A single nucleotide polymorphism (SNP) variant in the *DEFB101* promoter however, was shown to act protective in this setting (Kocsis et al, 2008). In line with the widespread expression of HBD1 and therefore its seemingly grave importance in epithelial defense, the variant also holds relevance in additional clinical contexts, *e.g*. in HIV transmission (Braida et al, 2004; Milanese et al, 2006) and oral infections (Jurevic et al, 2003). The SNP is generally functionally linked to HBD1 mRNA level, and interestingly also involved in the transcriptional expression of HBD3, at least in keratinocytes (Kalus et al, 2009). In the gut, and potentially also other tissues, *DEFB101* is furthermore partly under the control of nuclear receptor peroxisome proliferator-activated receptor (PPAR)γ (Peyrin-Biroulet et al, 2010), an essential mediator for intestinal homeostasis in response to dietary signals, inflammation and microbiota (Desreumaux et al, 2001; Marion-Letellier et al, 2009; Pothoulakis, 2009; Wehkamp et al, 2003a). Intestinal HBD1 is moreover dependent on Hypoxia-inducible factor (Hif)-1α, which binds a specific response element in the gene's regulatory region (Kelly et al, 2013). Other tissue studies allowed the identification of yet other regulatory factors in *DEFB101* transcription. The oncogene PAX2 could be identified as a repressor for HBD1 in prostate cancer (Bose et al, 2009). Interestingly, high levels of glucose and activation of the insulin pathway have also been proposed to induce HBD1 in different *in vitro* experiments (Barnea et al, 2008; Malik & Al-Kafaji, 2007). The various reports on *DEFB101* expression provide a glimpse on how even a rather stably expressed AMP can be dependent on several distinctive regulatory mechanisms. Such multi-layered interconnections in the control of antimicrobial defense might not only complicate investigations on the genetic regulation of the respective factors but particularly also hamper mechanistic studies in disease settings.

## AMPs in maintaining the host–microbe-homeostasis

Epithelial AMPs provide an important component within a complex barrier defense system and their production and secretion is an ongoing as well as an on demand process. From mice models we can get an idea about their essential functions in fencing of commensal but also pathogenic microbes. An induction of extensive Paneth cell degranulation (an excessive release of antimicrobials) *via* TLR9 stimulation for example, protects mice against *Salmonella typhimurium* infection (Rumio et al, 2004) but a lack of Paneth cell α-defensin (termed cryptdins in mice) activating enzyme conversely promotes a high susceptibility to orally administered pathogens (Wilson et al, 1999). A lack of the antimicrobial cathelecidin (mCRAMP in mice or LL-37 in humans), which is upregulated during ileitis or colitis dependent on the level of inflammation (Koon et al, 2011; Kubler et al, 2009; Schauber et al, 2006), leads to more severe symptoms and mucosal disruption in dextran sodium sulphate (DSS) chemically induced mouse colitis (Koon et al, 2011). Interestingly, the different abnormalities in this DSS challenged ko model, such as an increase in cytokine production and apoptotic cells, or impaired mucus production, can be reversed by intrarectal administration of or gene therapy with mCRAMP (Tai et al, 2013). Mice, which are transgenic for human defensin 5 and accordingly exhibit reshaped antimicrobial activity, are on the other hand protected against infections (Salzman et al, [Bibr b148]). Significant changes in the composition of gut microbiota when Paneth cell α-defensin defense is either doctored or diminished, also demonstrates its *in vivo* homeostatic role towards the symbionts (Salzman et al, 2010). Interestingly a person's nutritional status and lifestyle might play a crucial role in antimicrobial defense. Malnutrition for example, which is linked to diarrhea and inflammation, increased intestinal permeability and translocation of bacteria, has also been associated with differences in AMP expression and a dysbiosis of resident microbiota in the gut (Hashimoto et al, 2012; Hodin et al, 2011). Of special interest might be the fact that Paneth cells seem to be particularly affected by a lack of nutrition and show reduced AMP expression and granule abberations in a starvation mouse model (Hodin et al, 2011). This link between AMPs and intestinal barrier effectiveness did also become evident in a rat model of liver cirrhosis. Translocation of common intestinal microbiota could thereby be related to diminished epithelial α-defensins in the respective animals (Teltschik et al, 2011). Another study utilizing a mouse model of graft versus host disease (GVHD) also linked diminished Paneth cell antimicrobials to an increase of normally rare septicemia causing *E. coli* in expense of symbiotic diversity (Eriguchi et al, 2012). Of note, when comparing data from different mouse strains, one has to consider how a specific genetic background might differentially impact antimicrobial defense. C57BL/6 (B6) mice for example, exhibit 5 strain specific Paneth cell α-defensins, which have not been identified in other inbred mice (Shanahan et al, 2011). How this might be of general consequence in comparing results obtained in different models with otherwise seemingly comparable gene knockout approaches is easily imaginable. A study by Willing et al nicely employed how different mouse strains exhibit varying susceptibility to *Citrobacter rodentium* (a model of human pathogenic *E. coli* infections). They found that the different genetic background in investigated strains amass in variability in the architecture of the intestinal microbiotial community. The fact that transplantation of gut microbiota between strains promoted differentially activated innate defenses that could in turn partly abolish the symptom variability additionally nicely illustrates the reciprocity between host defense and symbiotic microbes (Willing et al, 2011). Such interplay might also be important in humans, for example in *Clostridium difficile* infections, where the transplantation of microbiota, or more precisely faecal transplants from healthy donors, have proven efficient in eradicating the pathogen and infection associated symptoms in patients (Borody & Khoruts, 2012). Faecal transplantation (FT) has also been introduced in other intestinal disorders, such as pseudomembranous colitis or irritable bowel syndrome amongst others, but large data sets on the different applications are still missing. Reports are often heterogeneous in the type of disease which was treated, what donors were used, as well as in their methodology and how they define a treatment response (Landy et al, 2011). Larger and randomized-controlled trials in addition to long-term follow-up studies are needed to gain evidence based insights on the procedure and its application before recommending faecal transplantation in a wider range of patients and disorders (Kassam et al, 2013; Khanna & Pardi, 2012). Effects of faecal transplantations on the microbiota compositions are yet also not fully clarified. Shifts from a disease related dysbiosis towards a healthier state in recipients have been reported but these often relate to quite small collectives, which are limited to *C. difficile* infected patients (Grehan et al, 2010; Khoruts et al, 2010). Recent works in this context reported high responding rates to FT, as well as a shift from a previous overabundance of *Proteobacteria* to a more normal composition of increased faecal bacterial diversity, similar to healthy donors, with an increase in *Bacteroidetes* (Shahinas et al, 2012; van et al, 2013). How long-standing such effects are and if and how FT affects the microbial ecology in the intestine of patients with other intestinal disorders is yet to be studied. Since the hosts genetic composition is also dramatically involved in shaping its microbiota, FT might be less effective in patients whose symptoms are not linked to a pathogenic catalyst but rather involve genetic susceptibility.

As mentioned, host AMPs for example exert a major influence on shaping microbial communities in the gut. It is therefore not surprising that the ability to evade them provided certain pathogenic strains with a selective evolutionary advantage (Gruenheid & Le, 2012; Koprivnjak & Peschel, 2011). This becomes clear in the case of *Helicobacter pylori*, which utilizes host cholesterol to gain resistance against cathelicidine (LL-37) (McGee et al, 2011). Furthermore, even though this gastritis promoting bacterium induces HBD2, it seems to selectively block another β-defensin, HBD3 (DEFB103) (Bauer et al, 2013). β-defensins share, like most AMPs, common characteristic biochemical properties like a positive charge and disulphide bonds that are important for their antimicrobial function (Boman, 2003). Even though these properties are quite similar between the respective HBDs, there can be vast differences in their effectiveness against different bacterial strains and in their modes of action. A striking example is provided by HBD1, for which earlier studies only reported weak antimicrobial function as compared to HBD2 or other defensins. The reason for this perceived lack of function became evident in work by Schroeder et al. The quite strong activity of HBD1 against various commensal bacteria and also a facultative pathogenic fungus depends on a biochemical activation in a reducing environment (Schroeder et al, 2011a,2011b). The ‘from zero to hero’ story of HBD1 highlights how often artificial experiment settings might miss important conclusions on antimicrobial efficacy. It is thus likely that additional capacities of defensins and other antimicrobials have yet to be elucidated and that the experimental environment might become a determining factor in this context. Aside from HBD1, HD6 also represents an exception of the antimicrobial activity rule. As mentioned earlier, it likewise did not exhibit extensive anti-bacterial activity in original studies. Chu et al were recently able to demonstrate how this Paneth cell α-defensin nonetheless helps to keep the epithelium sterile. It is able to form structures, which do not directly kill microbes but can disable them in a trap like strategy. HD6 polymerizes and forms peptide nanonets around bacteria, which have the potential to prevent them from being translocating across the gut wall. This mechanism does not directly affect microbial viability but can have a huge impact on microbial infectivity. Chu et al also confirmed the inhibitory effect of HD6 on translocation events in a *Salmonella* infected HD6 transgenic mouse model. This model nicely displayed the nanonet formation *in vivo* and also underlines the critical importance that HD6 likely has in human intestinal barrier integrity (Chu et al, 2012).

## Crohn's disease and ulcerative colitis – AMPs in chronic inflammation

**Figure 2:**
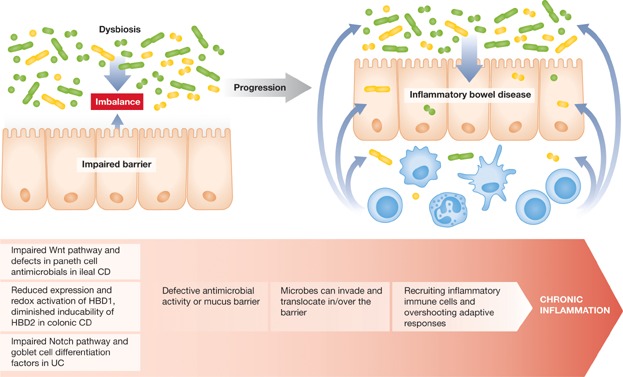
Proposed model for the pathogenesis of IBDs IBD is characterized by mucosal adherent bacteria and the induction of continuous and overshooting immune responses against normally commensal gut microbiota. Different defects in the intestinal barrier integrity, which affect the innate immune system, are linked to specific subgroups of IBD. Small intestinal CD is associated with defects in Paneth cell antimicrobial defense, which includes reduced α-defensin expression and Impairments in the Wnt pathway. In the colon, Crohn's disease is characterized by reduced inducability of HBD2 as well as diminished constitutive expression of HBD1 and the HBD1 reducing, and thereby activating thioredoxin. UC on the other hand shows an impaired inflammation associated induction of factors, which are important for goblet cell differenciation, which affects the mucus layer effectiveness. Such defects in the first line of mucosal defense can result in mucosal adherent bacteria as well as microbial translocations, which then promote ongoing inflammatory and adaptive immune responses.

In CD, a normally commensal or even mutualistic microbial community turns delinquent, leaving the host's immune system in a ‘with friends like these who needs enemies’ situation that subsequently promotes an ongoing inflammatory response (Fig 2). Recurring and grave intestinal inflammations in such affected patients are characterized by frequent diarrhea, ulcerations and sometimes multiple fistula (Podolsky, 2002). It is well accepted that to a similar degree both, genetic but also environmental factors contribute to the development of the disease (Halfvarson et al, 2006; Schreiber et al, 2005) but the mechanisms underlying the disturbed homeostasis are still extensively discussed. Epithelia of CD patients display mucosal adherent bacteria, activated T cells, and antibodies aimed not at specific pathogens but rather towards regular gut microbiota (Duchmann et al, 1999; Sartor, 1997). Researchers and clinicians therefore started to favour the idea of an epithelial barrier defect to rationalize this breech in homeostasis (Wehkamp et al, 2005a, 2008). In 2001, with the identification of the PRR *NOD2* as the foremost genetic CD susceptibility factor (Hugot et al, 2001; Ogura et al, 2001) the importance of innate defenses and a crucial role of disturbed bacteria-host interactions became commonly acknowledged.

It is important to address that CD symptoms can be quite heterogeneously in different patients, which might support distinguishing the disorder as a collection of similar but slightly different sub diseases. The Montreal classification for example phenotypes patients according to the affected gastrointestinal location as either solely small intestinal, exclusive colonic (and/or rectal) disease, or as a presentation with both, small and large intestine, involved. The patient's age of onset also plays an important part in subgrouping, as do the disease progression states which can range from a relatively weak ‘inflammatory’, to a more severe stenotic (with intestinal strictures), or a penetrating course with internal fistulae. Whereas behavioural characteristics might change over time, location maintains a relatively stable entity throughout the disease course arguing for differing pathogenesis mechanisms in small intestinal and colonic CD subgroups (Gasche & Grundtner, 2005; Louis et al, 2001; Silverberg et al, 2005). In the last years, peculiar defects in epithelial antimicrobial defense were identified which might not only explain the location specificity, but additionally accommodate an involvement of host impairments and the microbial component in disease development (Wehkamp et al, 2005a, 2008). Colonic CD is associated with an attenuated production of β-defensins *e.g*. reduced inducibility of HBD2 but also low HBD1 level, as mentioned previously. Since HBD2 can be induced by pathogens but also probiotic bacteria, *e.g. E.coli* Nissle but also certain *Lactobacilli via* the activation of NF-κB and activator protein 1 (AP-1) (Mondel et al, 2008; Ogushi et al, 2001b; Schlee et al, 2007; Wehkamp et al, 2003c, 2004a), a specific defect in β-defensin inducibility and/or function might likely explain why probiotic treatment seems to have promising effects in pouchitis and maintenance of remission in ulcerative colitis (UC, another IBD), but no benefit in CD (Schultz & Lindstrom, 2008). Further investigations on intestinal β-defensins might one day help to thoroughly understand their involvement in colonic CD disease aetiology.

The molecular mechanisms underlying another colonic IBD, UC, are also still under fervent discussion. The impact of genetics on the disease risk is with 20% comparably smaller and poorly understood environmental issues predominate with an 80% involvement in UC pathogenesis. Nonetheless similar to CD, bacterial contamination of the colonic mucus (Swidsinski et al, 2002) suggests that a host barrier problem is likewise a key problem in the disorder. Especially during inflammation, UC is associated with a thinner and in part even absent mucus layer (Pullan et al, 1994; Strugala et al, 2008) while defensins are readily induced (Wehkamp et al, 2003b). As mentioned, the ability of the mucus to bind and retain AMPs is crucial in protecting intestinal epithelia from bacterial adhesion and invasion (Meyer-Hoffert et al, 2008). It might therefore be that even though defensins are upregulated, the antimicrobial barrier is nonetheless severely compromised in UC. A clinical relevance of impairments in the intestinal epithelia covering mucus layer has also been confirmed in various models, which exhibited spontaneous or more severe DSS-induced colitis (Heazlewood et al, 2008; Johansson et al, 2011; Petersson et al, 2011). Furthermore, an inflammation associated and likely protective increase of mucus promoting goblet cell differentiation factors is found in CD, but absent in UC (Gersemann et al, 2009). One of the key regulators in this context, human atonal 1 (Hath1) might even be completely missing in affected colonic UC tissue (Zheng et al, 2011). In addition to such goblet cell differentiation impairments, another mechanism discussed in the context of UC associated mucus depletion centres on mucolytic gut microbiota. These bacteria are more frequent in IBD and might directly and negatively influence mucus thickness and stability (Png et al, 2010).

In the small intestinal presentation of IBD, ileal CD, a prominent reduction of constitutively expressed Paneth cell α-defensins HD-5 and -6 furthermore endorses a disease concept of defective antimicrobial defense (Wehkamp & Stange, 2010). Expression levels of a total of eight other Paneth cell products show no such decrease and the specific effect can also be seen independent from current inflammation. Of interest in this context is the fact that the association of *NOD2* shows specificity for the small intestinal disease subgroup and that the PRR is prominently expressed in Paneth cells. In 2010, Biswas et al used a *NOD-2* deficient mouse model to study the effect on Paneth cell defensins. When inoculated with the opportunistic pathogen *Helicobacter hepaticus*, a strong inflammatory response could be rescued by transgenic expression of HD-5 in *NOD2* deficient mice but not by a wild-type bone marrow transplant (Biswas et al, 2010). An *in vitro* model using one of the disease associated NOD2 variants, the frameshift mutation F3020insC, also underlined the PPRs important function in mediating antimicrobial defense (Begue et al, 2006). Whether disease relevant genetic variants of *NOD2* also precede even further reduced levels of Paneth cell α-defensins in humans, has been under ample discussion (Bevins et al, 2009; Simms et al, 2008; Wehkamp et al, 2004b, 2005b). A recent study in *NOD2* deficient mice on a BL6 background demonstrated only a reduced expression of cryptdin-related sequence 1C, but other Paneth cell antimicrobials were unchanged. The work by Shanahan et al also displayed how differences in faecal microbiota between the ko and wildtype mice were overwritten by cohousing the animals (Shanahan et al, 2013). If and how a change in mucosa adherent bacteria might still be present could be an interesting question in future research in this *NOD2* model. Not only the genetic background of laboratory mice influences epithelial antimicrobial defense (Gulati et al, 2012), but also their habitat exhorts a profound impact on gut microbiota (Ma et al, 2012). Since there are also profound differences between mice and humans regarding adaptive and innate immune functions in general (Mestas & Hughes, 2004; Seok et al, 2013), it is apparent how model systems might not always reflect the situation in patients who have defects in the studied genes. It is therefore essential for the study of NOD2 but also other factors in antimicrobial defense to follow-up on mechanistic studies in additional settings, *e.g*. primary, intestinal structure forming organoids from adult stem cells (Howell & Wells, 2011; McCracken et al, 2011).

Besides NOD2, also other CD pathogenesis involved factors and/or associated genes are proposed in the context of Paneth cell antimicrobial defense (Table 1). Similar to *NOD2*, the genetic association of ‘*ATG16 autophagy related 16-like 1*’ (*ATG16L1*) (Cadwell et al, 2008) shows an increased effect in the small intestinal subphenotype (Fowler et al, 2008; Hampe et al, 2007). The *ATG16L1* risk variant is accompanied by Paneth cell abnormalities in patients, which are correspondingly present in ATG16L1^HM^ mice (Cadwell et al, 2008). The bona fide autophagy protein has essential functions in granule exocytosis pathways and thus in peptide export from secretory cells. The maintenance of secretory cells as the AMP producing Paneth cell is also influenced by the CD associated *X-box binding protein 1* (*XBP1*) (Kaser et al, 2008), a key component of the endoplasmic reticulum (ER) stress response (Acosta-Alvear et al, 2007). Its deletion in mouse intestinal epithelia results in spontaneous enteritis and increased colitis susceptibility preceded by Paneth cell dysfunction amongst other epithelial impairments (Kaser et al, 2008). Another CD associated factor, the ‘*intermediate conductance calcium-activated potassium channel protein*’ (*KCNN4)* encoded K_Ca_3.1 protein has an important function in T cell Ca(2+) signalling and, like ATG16L1, a role in Paneth cell secretion. Besides its genetic association with the disease, *KCNN4* exhibits significantly reduced mRNA in *NOD2* mutated patients (Simms et al, 2010), highlighting a rather complex genetic interplay in the disease.

**Table 1:** Genetic associations in Crohn's disease with a relevance in the specialized antimicrobial producing Paneth cell

Factor	Full gene name	Core functions	Relevance in Paneth cell
Factors with a direct link to Paneth cell function			
*NOD2*/CARD15*	Nucleotide-binding oligomerization domain-containing protein 2/caspase recruitment domain-containing protein 15	Intracellular PRR sensing bacterial muramyldipeptide	NOD2 is involved in the expression of Paneth cell defensins and the activation of innate antimicrobial defense strategies (Begue et al, 2006). Carriers of a frameshift risk variant have been reported to exhibit particularly low Paneth cell α-defensin levels (Bevins et al, 2009; Wehkamp et al, 2005b).
Atg16L1*	Autophagy related 16-like 1 (*S. cerevisiae*)	Part of a protein complex involved in autophagy, the major degradation system of cytoplasmatic components	ATG16L1 is involved in the granule exocytosis pathway and respectively the secretion of Paneth cell AMPs. Patients carrying the associated risk variants display Paneth cell abnormalities (Cadwell et al, 2008)
*XBP1*	X-box binding protein 1	Important transcription factor in the ER stress response as well as secretory cell development and maintenance	XBP1 deletion results in apoptotic Paneth cell loss and reduced antimicrobial activity. In addition to the association of common SNPs, the gene also exhibits rare hypomorphic non-synonymus variants in IBD patients (Kaser et al, 2008)
LRP6*	Low density lipoprotein related receptor 6	Wnt Co-receptor, R-Spondin receptor and LGR interaction partner with an important role in β-catenin dependent Wnt	LRP6 expression levels are linked to those of Paneth cell HD5 *in vitro*. The receptor's mRNA is furthermore reduced in small intestinal CD and an early onset associated non-synonymous risk variant precedes even further reduced levels of HDs (Koslowski et al, 2012)
TCF7L2*	Transcription factor 7-like 2 (T-cell specific, HMG-box), also known as TCF4	Transcription factor and interaction partner of β-catenin. Important regulator of Wnt target genes	TCF7L2 is reduced in and genetically associated with small intestinal CD. It binds the promoter region of HD5/6 and regulates the α-defensins transcriptional expression (Koslowski et al, 2009b; Wehkamp et al, 2007)
**Factors with a hypothesized role in diminished Paneth cell function in CD patients**			
*Lef1*	Lymphoid enhancer-binding factor 1	Transcription factor and interaction partner of β-catenin. Important regulator of Wnt target genes and associated with CD (Dinu et al, 2012). It's role in canonical Wnt would support a potential involvement in Paneth cell function and in particular in the regulation of the α-defensins HD5 and HD6	A CDH1 CD risk haplotype precedes increased cytoplasmic E-cadherin likely due to a truncated form of the protein. This protein version also promotes impaired β-catenin localisation *in vitro* and might therefore be relevant for the canonical Wnt activity in Paneth cells (Muise et al, 2009)
*CDH1*	Cadherin-1 or epithelial cadherin (E-cadherin)	A calcium-dependent cell–cell adhesion glycoprotein involved in mechanisms regulating epithelial cell adhesion, mobility and proliferation	
KCNN4*	Potassium intermediate/small conductance calcium-activated channel, subfamily N, member 4	Part of a voltage-independent potassium (K(+)) channel activated by intracellular calcium (Ca(2+))	The genetically associated KCNN4 encodes KCa3.1, which is found in Paneth cells. NOD2 risk variant carriers also exhibit reduced KCNN4 mRNA (Simms et al, 2010). In mice, a Ca(2+)-activated K(+) channel modulates Paneth cell secretion (Ayabe et al, 2002) which might allow to hypothesize a similar relevance of KCa3.1 in humans

* Associations are known to be stronger or specific to the small intestinal Crohn's disease subphenotype.

A new view on CD is based on an involvement of disturbed Wnt signalling in the small intestinal disease subgroup. The Wnt pathway is an important regulator of epithelial proliferation and interestingly also Paneth cell maturation (Crosnier et al, 2006). The β-catenin dependent Wnt cascade (also called ‘canonical’), depends on activation of Frizzled and ‘low density lipoprotein receptor-related protein’ (LRP) 5 or 6 receptors by Wnts and subsequent accumulation of cytoplasmatic β-catenin. In the absence of Wnt, β-catenin is associated with a complex containing adenomatous polyposis coli (APC), glycogen synthase kinase 3 β (GSK-3β) and axin amongst others (Aberle et al, 1997; Fagotto et al, 1999; Orford et al, 1997). This destruction complex mediates GSK3β- dependent phosphorylation and subsequent ubiquitination-dependent proteasomal degradation of β-catenin. Inhibition of the formation and/or activity of the destruction complex upon receptor ligand interaction promotes accumulation of β-catenin. The central pathway component can then enter the nucleus and activate target gene expression in cooperation with transcription factors of the ‘lymphoid enhancer-binding factor’ (Lef)/‘transcription factor (T-cell specific, high-mobility group (HMG)-box)’ (TCF) family. Multiple studies could show a critical Paneth cell dependence on canonical Wnt (Andreu et al, 2005, 2008; van Es et al, 2005, 2012). In small intestinal CD, reduced expression (Perminow et al, 2010; Wehkamp et al, 2007; Zilbauer et al, 2011) and a genetic association (Koslowski et al, 2009b) of *TCF7L2* as well as an early onset associated coding variant in *LRP6* and generally low mRNA level of the Wnt co-receptor (Koslowski et al, 2012), support an important role of the pathway in disease aetiology. Furthermore, *E-cadherin* (*CDH1*) which has also been introduced in the context of CD susceptibility provides an additional bridge to disturbed canonical Wnt activity (Muise et al, 2009). Even though it has not directly been linked to Paneth cell function, its roles in β- catenin localization would support an influence on canonical signalling activity and subsequently the cell's maturation and gene program. Another indirect hit in the β-catenin dependent cascade was provided by a recent study using logic regression to reevaluate genetic data from a genome wide approach (Dinu et al, 2012). The used innovative statistical technique allows the selection of a model, which potentially involves multiple intersections and/or unions of SNPs within a certain gene, or any set of SNPs (*e.g*. various genes which are relevant in a specific pathway), that are associated with the phenotype of interest. In the analysis, the canonical Wnt signalling transcription factor *LEF1* was found to be amongst the genes with the strongest evidence for an association with the risk of CD. Impaired cell proliferation or differentiation as a novel pathogenesis concept might provide promising although complex opportunities for new and causal therapeutic intervention. In addition, studies on the interplay of the different Paneth cell affecting genetic hits and integrating the role of microbiota in this context could improve our understanding of symptomatic heterogeneity or disease severity and might one day assist in identifying critical risk patients.

## The epithelial regenerative homeostasis

**Figure 3:**
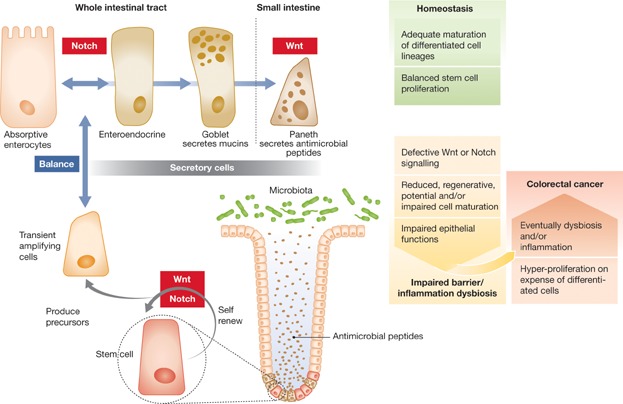
The gut is characterized by a delicately balanced regeneration Intestinal epithelia underlie complex signalling networks, first and foremost the Notch and Wnt pathways, which control the symmetry of proliferation and differentiation. Notch furthermore directs the lineage decision between absorptive and secretory cells, while the β-catenin dependent Wnt cascade additionally regulates Paneth cell maturation and function. Different from all other secretory cell types (Enteroendocrine and Goblet cells), Paneth cells are normally confined to the bottom of small intestinal crypts of Lieberkühn but can under certain circumstances (*e.g*. inflammation) appear in other gastrointestinal epithelia. A disturbance of the epithelial cell homeostasis might have disastrous consequences promoting an either debilitated barrier or potentially tumour development. In both cases, effects of microbiota on the onset and/or progression of subsequent pathogenesis mechanisms are discussed. Bacteria have also been shown to influence epithelial differentiation factors in general, which highlights a circular symmetry of host microbe relationship in the gut.

The human intestinal epithelial lining undergoes cell renewal at an extraordinary rate much faster than any other tissues in our body (Gregorieff & Clevers, 2005). All present cell types descend from multipotent stem cells located at the base of the crypts, right above and/or between the Paneth cells (Fig 3). They self-renew and generate an adjacent zone of rapidly cycling progenitors, which again increase their pool before differentiating into multiple lineages, creating up to 300 cells/cryp/day (Barker et al, 2008). The post-mitotic crypt necks and villus regions make up the biggest part of the intestinal epithelium (Crosnier et al, 2006) whereas Paneth Cells escape the upwards flow and reside at the crypt base for 3–6 weeks (Barker et al, 2008). As mentioned, proliferation is in large parts subject to the activity of Wnt (Korinek et al, 1998) but it is also dependent on Notch signalling (Fre et al, 2009). Both signals, Wnt and Notch are paradoxically also essential for the directed differentiation of specific cell types, Notch for the secretory lineage decision and Wnt for Paneth cell maturation (Jensen et al, 2000; van Es et al, 2005). In this context, the propable stem cell marker ‘leucine-rich repeat G protein-coupled receptor’ (LGR5) (Barker et al, 2007) also has important functions in Paneth cell maturation. It's deficiency results in premature Paneth cells but generates no major defects in other lineages, progenitor proliferation or cell migration in the small intestine (Garcia et al, 2009). Data on LGR4 show similar effects on gut homeostasis, it's disruption in mice leads to diminished Paneth cell numbers as well as increased DSS colitis severity (Liu et al, 2013). Recently two groups published almost simultaneously, how LGR5 and its homologues, like LGR4, can bind R-Spondins associate with Frizzled/LRP and potentiate Wnt-activity (Carmon et al, 2011; de et al, 2011) which nicely explains their importance for Paneth cell biology.

Even though intestinal proliferation pathway networks are quite complex, one needs to consider that they are also additionally subjected to various influences from innate immune and inflammatory signalling. The host–microbe homeostasis can hence be relevant for epithelial homeostasis and *vice versa* since epithelia on their hand can, *e.g. via* AMP production, control the composition of gut microbiota. It was recently shown that Lgr5+ putative intestinal stem cells express TLR4, which, when activated *via* bacterial LPS, promotes apoptosis while reducing *in vivo* proliferation in mice (Neal et al, 2012). In the context of necrotizing enterocolitis (NEC), the leading cause of death from gastrointestinal disease in preterm infants, the role of TLR4 has furthermore been studied in mice models and *in vitro* experiments. In both settings, TLR4 stimulation promoted β-catenin inhibition *via* GSK3β activation cumulating in reduced cell proliferation rates (Sodhi et al, 2010). The same group also followed up with functional studies on the effects of TLR4 in intestinal epithelia *via* deletion of the PRR in mice and cell culture. This deletion diminished Notch pathway activity and increased goblet cell numbers in the small intestine of mice and complementarily augmented goblet cell differentiation in cultured enterocytes (Sodhi et al, 2012). Another TLR4 mediated mechanism with a role in NEC was recently identified in the context of epithelial autophagy. TLR4 activation induced autophagy in enterocytes, which was a requirement for and not only a consequence of NEC development in the studied mouse model (Neal et al, 2013). Different from the proliferation inhibiting effects of TLR4, investigations on TLR2 and TLR5 implicated them in promoting epithelial repair, growth and survival. The observed effects of those PRRs were not linked to the release of cytokines after TLR stimulation, but rather to the activation of different receptor tyrosine kinases including members of the epidermal growth factor receptor (EGFR) family (Shaykhiev et al, 2008). The setting of the respective work focused on airway epithelial cells but since both receptors have also been implicated in hyper-proliferation of gastric epithelia (Song et al, 2011; Tye et al, 2012), this mechanism might also be relevant in the gut. An influence of TLR9 on gut epithelial homeostasis has also been investigated in ko mice. Respective animals display increased numbers of proliferating cells while levels of the Notch target gene hairy enhancer of split 1 (hes1), a differentiation factor directing cells towards the enterocyte fate, and vascular endothelial growth factor (VEGF), which is important for epithelial cell restitution, were reduced. TLR9-deficient mice were furthermore highly susceptible to DSS induced inflammation and exhibited delayed wound repair (Rose et al, 2012). Complementing this observation, an influence of gut bacteria on the transcription of the Notch target Hes1 and other differentiation factors has also been proposed (Becker et al, 2013). The early determination of secretory versus absorptive cell fate is regulated by an antagonistic interplay of Hes1 and the basic helix–loop–helix transcription factor Hath1. Progenitor cells expressing Hes1 block Hath1, which favours the absorptive lineage, while an inactive Notch/Hes1 signalling allows Hath1 transcription and a transit towards a secretory fate. Cells entering the secretory line require additional signals to mature into specific cell types such as, for goblet cells, the zinc-finger transcription factor krüppel like factor (KLF)4 or as mentioned for Paneth cells, active β-catenin. *In vitro* expression of Hes1, Hath1, and to a minor degree also of KLF4, can be reduced by a pathogenic or probiotic *E. coli*. Accommodatingly, germ free mice also display diminished colonic expression of Hath1 and KLF4 in comparison to specific pathogen free or conventionalized counterparts, even though a direct effect on goblet cell numbers cannot be observed (Becker et al, 2013). Besides bacteria, other intestinal inhabitants can also influence epithelial proliferation and differentiation. Mice infected with *Trichinella spiralis*, a parasite that drives small intestinal inflammation, show changes in mucosal architecture, *e.g*. an elevated amount of proliferative progenitors and Paneth cells (Walsh et al, 2009).

The relationship between microbes, innate immune and inflammatory pathways, and cell proliferation seems to be quite complicated and complex but even more, it is also dual. β-catenin itself functions as a constitutive negative regulator of *in vivo* inflammation. Similar to the inhibitor IkB, the central canonical Wnt pathway component can bind NF-κB and can prevent its activity (Duan et al, 2007). In response to pathogenic (*Salmonella*, *Yersinia*, *Listeria* and enteropathogenic *E. coli* (EHEC)) but not non-virulent bacteria, the physical interaction between the two signalling components is compromised subsequent to GSK3β dependent β-catenin degradation. Conversely, a study in colon and breast cancer cells could demonstrate that GSK3β inhibition can also alter NF-κB activity through β-catenin stabilization, which again links canonical Wnt with inflammatory signalling (Deng et al, 2004). Phosphorylation of GSK3β and β-catenin stabilization seem to provide important control points in inflammatory processes, suggesting that activated β-catenin may be a balancer of bacteria-induced inflammation in general and likely also in the gut. A failure of the system could be fatal, shifting epithelial immune reactions towards a more inflammatory status. In monocytes such a pivotal role for GSK3β has been demonstrated in deciding inflammatory responses after TLR activation (Martin et al, 2005). In addition, it has also been reported that dendritic cells require β-catenin-dependent signalling to mediate gut tolerance to commensal microbes in mice. Ablation of β-catenin in dendritic cells reduced regulatory T cells and anti-inflammatory cytokines while increasing pro-inflammatory processes promoting an enhanced susceptibility to experimental colitis in this model (Manicassamy et al, 2010). For the epithelial setting it has furthermore been shown that levels of Wnt2, a ligand in the canonical cascade, are elevated after bacterial infection. An *in vitro* knock down of Wnt2 enhances bacteria induced epithelial IL8 expression, which is conversely less secreted in Wnt2 overexpressing cells (Liu et al, 2012). Upregulation of Wnt2 might hence be a host strategy to inhibit an overshooting inflammatory response during infection by activation of β-catenin.

Multiple crosstalks between proliferative, immunological and inflammatory pathways complicate the picture of epithelial homeostasis in the gut. This is especially tricky when considering tampering with specific pathways to test their therapeutic value. Blocking a signalling cascade might on the one hand dampen pro-inflammatory processes but might on the other hand impact epithelial differentiation. An example for this scenario is provided by TLR4. The PRR is upregulated during intestinal inflammation (Hausmann et al, 2002) and can induce autophagy in enterocytes (Neal et al, 2013). Blocking the PRRs with an antibody in fact ameliorates inflammation but unfortunately also delays mucosal healing in DSS treated mice (Ungaro et al, 2009).

## Colorectal cancer – a role for microbes and AMPs

Colorectal cancer (CRC) has recently been linked to a dysbiosis of gut microbiota (Sobhani et al, 2011). In their study, Sobhani et al identified 18 genera with an abundance of more than 1%. Thirteen of these genera (*Alistipes, Collinsella, Bacteroides, Lachnospira, Prevotella, Subdoligranulum, Dorea, Faecalibacterium, Roseburia, Coprococcus, Streptococcus, Bifidobacterium* and *Ruminococcus*) corresponded to the previously described human intestinal microbiota phylogenetic core of which certain species were also specifically shown to be decreased in cancer patients: *Bifidobacterium longum*; BG; AY675246, *Clostridium clostridioforme*; 1-53; AY169422, *Ruminococcus sp. DJF_VR66*; EU728790, *Ruminococcus bromii*; L2-63; EU266549. Their analysis furthermore showed that more than 7% of the microbiota variability was impacted by the cancer status. This particular study could also connect variations in the distribution of bacterial genera in faecal samples with the disease status, and demonstrated a significantly elevated *Bacteroides/Prevotella* population, which correlated with an increase of IL-17 in mucosal samples of respective patients. A more recent work by Ohigashi et al also consistently reported a divergence in faecal microbiota as well as a significant decrease of the concentrations of short chain fatty acids (SCFAs) in a similar setting and furthermore reported additional differences in the intestinal environment, including an elevated pH (Ohigashi et al, 2013). Another study in six CRC patients did not focus on faecal microbiota but rather elucidated how tumour tissue directly seemed to harbour a specific microbiome which was strikingly different from adjacent non-malignant mucosa in five of the investigated CRC patients (Marchesi et al, 2011). The progression of colorectal cancer is coupled to deregulation of different signals and pathways, amongst others, an augmentation of canonical Wnt, often mediated by deactivating mutations in APC (Goel & Boland, 2010; Saleh & Trinchieri, 2011; Vaiopoulos et al, 2012). Proinflammatory cytokines have been shown to enhance Wnt β-catenin/TCF transcriptional activity in this setting and patients with colonic IBD have been shown to bear an increased risk for CRC development in epidemiological studies (Saleh & Trinchieri, 2011). The cytokine IL-1β for example can promote GSK3β inactivation and subsequent β-catenin stabilization (Kaler et al, 2009). An impact of microbiota in this context has also been investigated. In azoxymethane (AOM), chemically induced inflammation of conventional, *Bacterioides vulgatus* mono-associated, and germfree IL-10(−/−) and Myd88(−/−) mice, it could be shown that the risk for colitis associated cancer seems to be TLR/MyD88 dependent and can be altered by manipulation of intestinal microbes (Uronis et al, 2009). In a similar setting, Arthur et al could demonstrate that specific bacterial abilities can mediate cancer promoting effects and argued that colitis may foster the expansion of microbes with such deleterious capabilities (Arthur et al, 2012). In their study, commensal *E. coli NC101* promoted CRC in AOM treated IL-10-deficient mice while a deletion of the bacterium's polyketide synthase island decreased cancer multiplicity and invasion without affecting the inflammation in the model. CRC is not the only gastrointestinal malignancy with a connection to bacteria induced inflammation. Deregulation of NF-κB and canonical Wnt is also present in a majority of gastric cancers (Ooi et al, 2009) and induction of high nuclear β-catenin by *H. pylori*, a major cause of gastric malignancies, provides again, a link to bacteria. Besides the hosts genetic predisposition, *H. pylori*'s capacity to inhibit GSK3β activity is likely responsible for the outcome and persistence of intestinal metaplasia in gastritis (Hung et al, 2009). Intestinal colonization by another *Helicobacter* species, *H. hepaticus*, furthermore activates NF-κB-regulated networks both in the colon but also in liver and promotes hepatic cancer marked by canonical Wnt activation without bacterial translocation or hepatitis induction in mice (Fox et al, 2009).

Since AMPs are critical in controlling the enteric microbiota, they might well have a role in bacteria driven cancer development. Unfortunately this arc in the story of microbial influenced colonic cancer has not yet been investigated in detail. Other functions of AMPs however, have already been studied in this context. In addition to directly killing pathogens or regulating enteric symbionts, AMPs have a role in orchestrating adaptive immune responses. Some have chemotactic ability to recruit immune cells from monocytic and lymphocytic lineages (Lai & Gallo, 2009). HBDs have for instance been shown to recruit immune cells (*e.g*. monocytes, macrophages, and neutrophils but also T-cells) to the site of microbial invasion through interaction with the CC-chemokine receptor (CCR)6 and CCR2 (Rohrl et al, 2010a,2010b; Yang et al, 1999). CCR dependent mBD14 (the mouse ortholog of HBD3) recruitment of macrophages has been shown to indirectly promote tumourigenesis *via* the induction of pro-inflammatory cytokines (Rohrl et al, 2012a,2012b). Since *in vitro* results and data from mice models on the other hand also show anti-inflammatory influences of HBD3 on monocytes and macrophages (Semple et al, 2010, 2011), its role in inflammation promoted cancer could however be more complex. If and how the manipulation of AMP expression or a direct alteration of gut microbiota might be a preventative strategy in inflammation induced colon cancer, provides nonetheless interesting questions for supplementary future research.

**Pending issues**

Mice models of inflammation have more and more been shown to bear a grave divergence from human immune processes. The limitation is especially high regarding Paneth cell antimicrobials and is not only linked to genetic differences (eg expression of a differing set of α-defensins) but can depend on factors like nutrition or housing. It will become increasingly essential to follow-up on mechanistic studies in additional settings, *e.g*. primary organoids from human adult stem cells, which are just now emerging.

AMPs are crucial in the regulation of gut microbiota. Chronic intestinal inflammation as well colon cancer has been associated with a dysbiosis. If and how the manipulation of AMPs or a direct alteration of gut microbiota might be a preventative strategy in chronic inflammation and inflammation induced colon cancer has yet to be determined. It might provide interesting questions for future research and new therapy approaches.

Faecal transplantation (FT) can be successfully used in persistent *C. difficile* infections and has been linked to microbiota shifts in the recipients. How long-standing these are, and if and how they might occur in other intestinal disorders are not yet clear. Since the hosts genetic make-up is dramatically involved in shaping its microbiota, FT might be less effective in patients with symptoms linked to genetic susceptibility rather than to a microbial pathogenci cause. Future studies might provide valuable new information on the application possibilities of FT as well as the determining factors of host–microbe homeostasis in different diseases.

## Conclusions

As we highlighted in this review, the interconnections between AMPs, gut microbiota, innate immune signalling and epithelial proliferation pathways are quite complex. Disturbed antimicrobial function in the gut can provoke devastating consequences on barrier effectiveness and likely promotes a shift in the microbial community. In addition, an altered composition of gut microbiota can on its part influence and destabilize epithelial defenses. Such breeches of homeostasis on either the host or the microbiota side likely promote a vicious self-feeding cycle. A similar hostile chain of events could be enabled during the course of IBD or might even play a role in inflammation associated cancer risk. Further detailed investigations on the interplay of gut epithelial host–microbe interactions, inflammation and proliferation pathways are essential to complete our understanding in that matter. Such integral approaches are ambitious; nonetheless accepting this challenge holds promises for the development of new and causal therapeutic avenues in different intestinal disorders.

Conflict of interest statement: Dr. M. Ostaff (née Koslowski), Prof. Dr. EF Stange and PD Dr J. Wehkamp are named inventors on the patent application: WO2010072389, a method for determining the predisposition for Crohn's disease, developed in their lab. Prof. Dr. EF Stange and PD Dr J. Wehkamp are furthermore named inventors on WO 2012/028479: Material combination for treating inflammatory or infectious diseases, which was also developed in their lab.
